# Supplementary material for: Targeting Lactate Dehydrogenase A with Catechin Resensitizes SNU620/5FU Gastric Cancer Cells to 5-Fluorouracil
Source: Int J Mol Sci. 2021 May 20;22(10):5406. doi: 10.3390/ijms22105406 (PMC8161398; doi:10.3390/ijms22105406)
Supplement: Supplementary file 1 [file ijms-22-05406-s001.zip › ijms-1199528-supplementary.pdf]

International Journal of
Molecular Sciences

Supplementary Methods

1.1. Cell Counting

The growth of SNU620 and SNU620/5FU were estimated using cell counting. The cells were seeded in 24 well plates (5 X 10^4 cells/well) and cultured for the stated day. Cell counting was conducted by mixing 10 μl of cells and 10 μl of trypan blue for the exclusion of dead cells. The cells were counted on a hemacytometer using a microscope.

1.2. Isothermal Titration Calorimetry (ITC) Analysis

ITC analysis was using MicroCal auto_iTC200 (GE Healthcare). The purified LDHA protein and CA were dialyzed in the same buffer. The buffer composition is 200 mM NaCl and 50 mM Tris-HCl (pH 8.0) at a concentration of 0.1 mM. CA solution was titrated with an LDHA solution against the ITC200 microcalorimeter cell. These raw data peaks were transformed and normalized using the MicroCal Origin 7.0 software (GE Healthcare) to obtain a stoichiometry (N) binding affinity (K), enthalpy (ΔH), and entropy (ΔS).

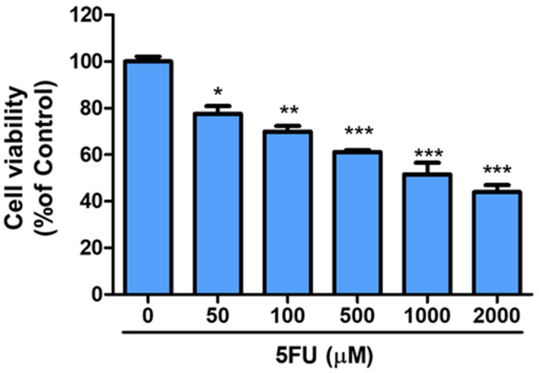

Supplementary Figure S1. Effect of 5FU on the viability of SNU620/5FU cells. The cells were treated with the indicated concentration of 5FU for 2 days. After incubation, the viabilities of cells were measured by MTT assay. The results are shown as mean ± SEM. * $p < 0.05$, ** $p < 0.01$, and *** $p < 0.001$ compared to the control.

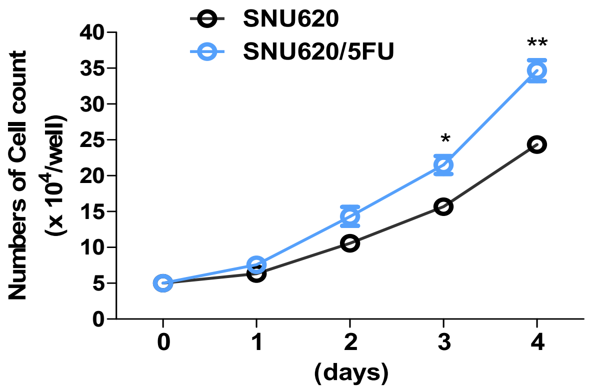

Supplementary Figure S2. The growth of SNU620 and SNU620/5FU cells measured by cell counting. The 5 X 10^4 cells were seed in 24 well plates. The cells were incubated until 4 days, and the numbers of cells were measured at each day by hemacytometer cell counting. The results are shown as mean ± SEM. * $p < 0.05$ and ** $p < 0.01$ compared with the respective control.

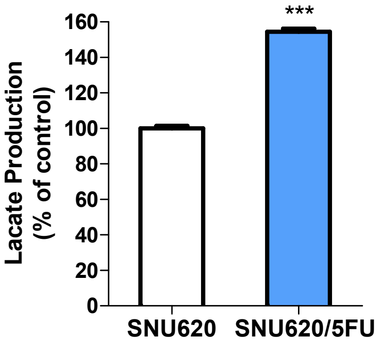

Supplementary Figure S3. Lactate production levels of SNU620 and SNU620/5FU cells. The cells were incubated for 1 day at 37°C, after which the culture medium was replaced with phenol red-free medium and incubated for 1 h at 37°C. Following incubation, the medium of each cell was assessed using a commercial lactate fluorometric assay kit. The results are shown as mean ± SEM. *** p <0.001 compared to the control.

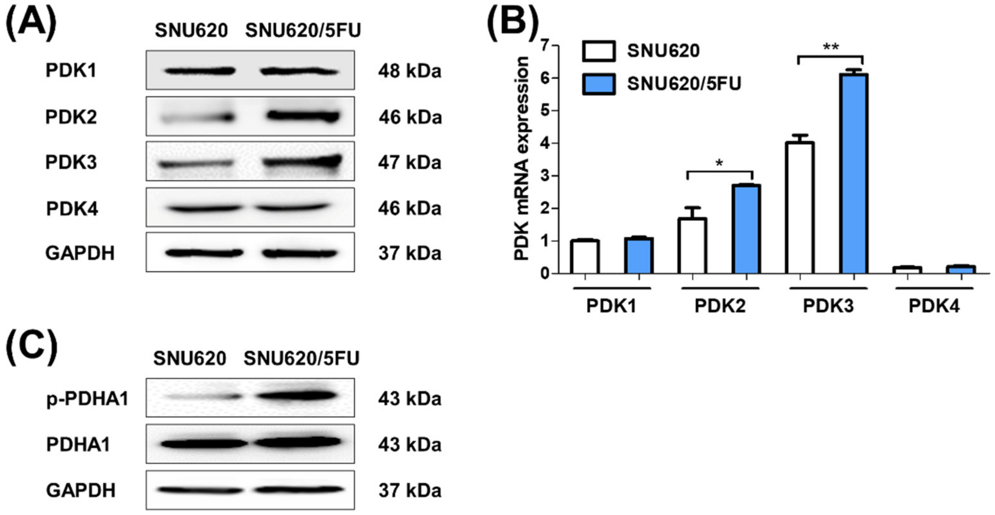

Supplementary Figure S4. Expressions of PDK isotypes in SNU620 and SNU620/5FU cells. The proteins and mRNAs were extracted from the cell lysates of SNU620 and SNU620/5FU cells incubated for 1 day at 37°C. **(A)** The protein expression of PDK1, PDK2, PDK3, PDK4, and GAPDH were measured via western blot analysis. **(B)** The mRNA expression levels of PDK1, PDK2, PDK3, and PDK4 were evaluated via qRT-PCR. The results are shown as mean ± SEM. * $p < 0.05$ and ** $p < 0.01$ compared to the respective control value. **(C)** The expression levels of p-PDHA1, PDHA1, and GAPDH were detected via western blot analysis.

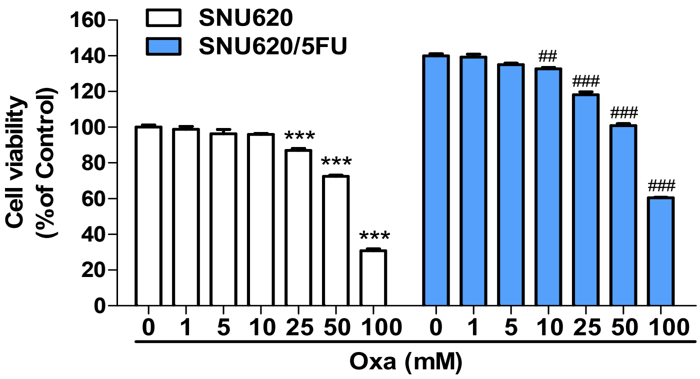

Supplementary Figure S5. Effect of oxamate on the viabilities of SNU620 and SNU620/5FU cells. The cells were treated with the indicated concentrations of oxamate. After 48 h, the viabilities of these cells were measured via MTT assay. The results are shown as mean ± SEM. *** $p < 0.001$; ## $p < 0.01$ and ### $p < 0.001$ compared with the respective control.

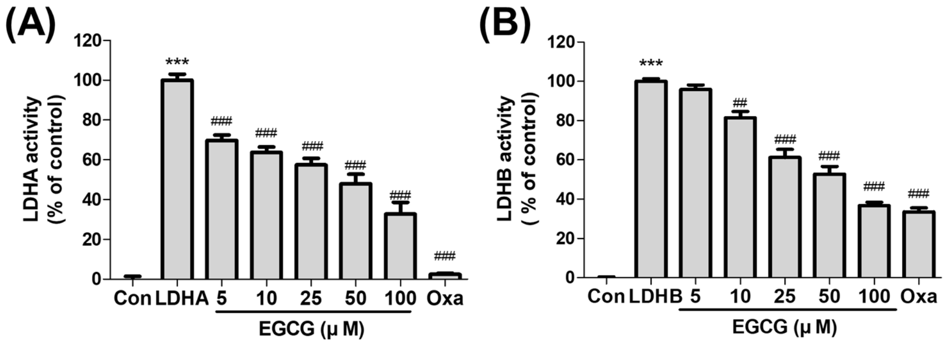

Supplementary Figure S6. Effects of EGCG on LDHA and LDHB activities. **(A, B)** LDHA and LDHB activities were measured via *in vitro* LDH activity assay using recombinant LDHA and LDHB proteins. The indicated concentrations of EGCG were incubated with LDHA or LDHB protein with buffers and substrates (NADH for LDHA and NAD^+ for LDHB). Fluorescence of NADH with an excitation wavelength of 340 nm and an emission wavelength of 460 nm was detected using a spectrofluorometer. The results are shown as mean ± SEM. *** $p < 0.001$ compared to the control (1st column). ## $p < 0.01$ and ### $p < 0.001$ compared to the negative control (2nd column).

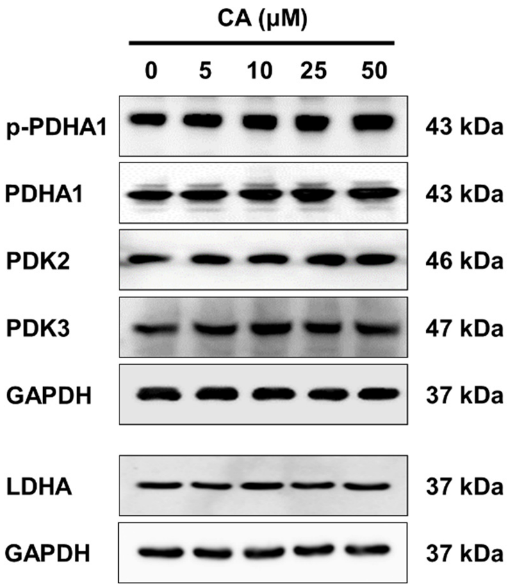

Supplementary Figure S7. Effects of catechin (CA) on the expression levels of p-PDHA1, PDHA1, PDKs, and LDHA proteins. SNU620/5FU cells were treated with the indicated concentrations of CA for 24 h. The cell lysates were used for determining the expression levels of p-PDHA1, PDHA1, PDK2, PDK3, LDHA, and GAPDH via western blot assay.

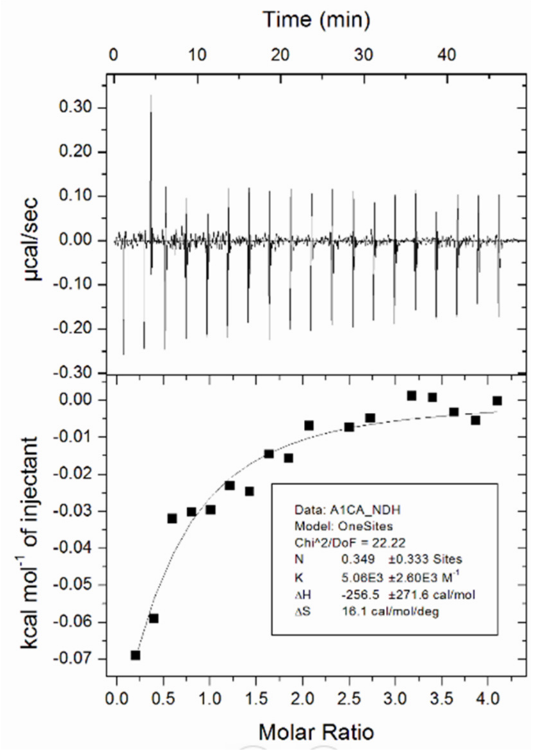

Supplementary Figure S8. Isothermal titration calorimetry (ITC) analysis of the LDHA and CA. Data for the titration of CA with LDHA.

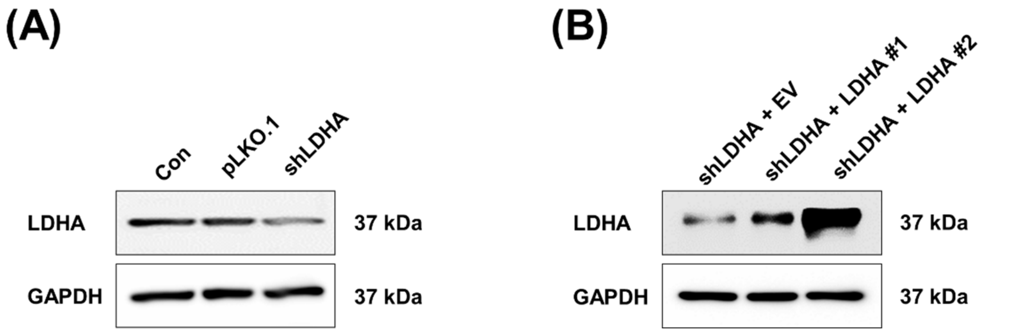

Supplementary Figure S9. LDHA expression in SNU620/5FU cells transfected with shLDHA. **(A)** or LDHA overexpression vectors **(B)**. The cell lysates were obtained and protein levels of LDHA and GAPDH were examined by Western blot analysis.

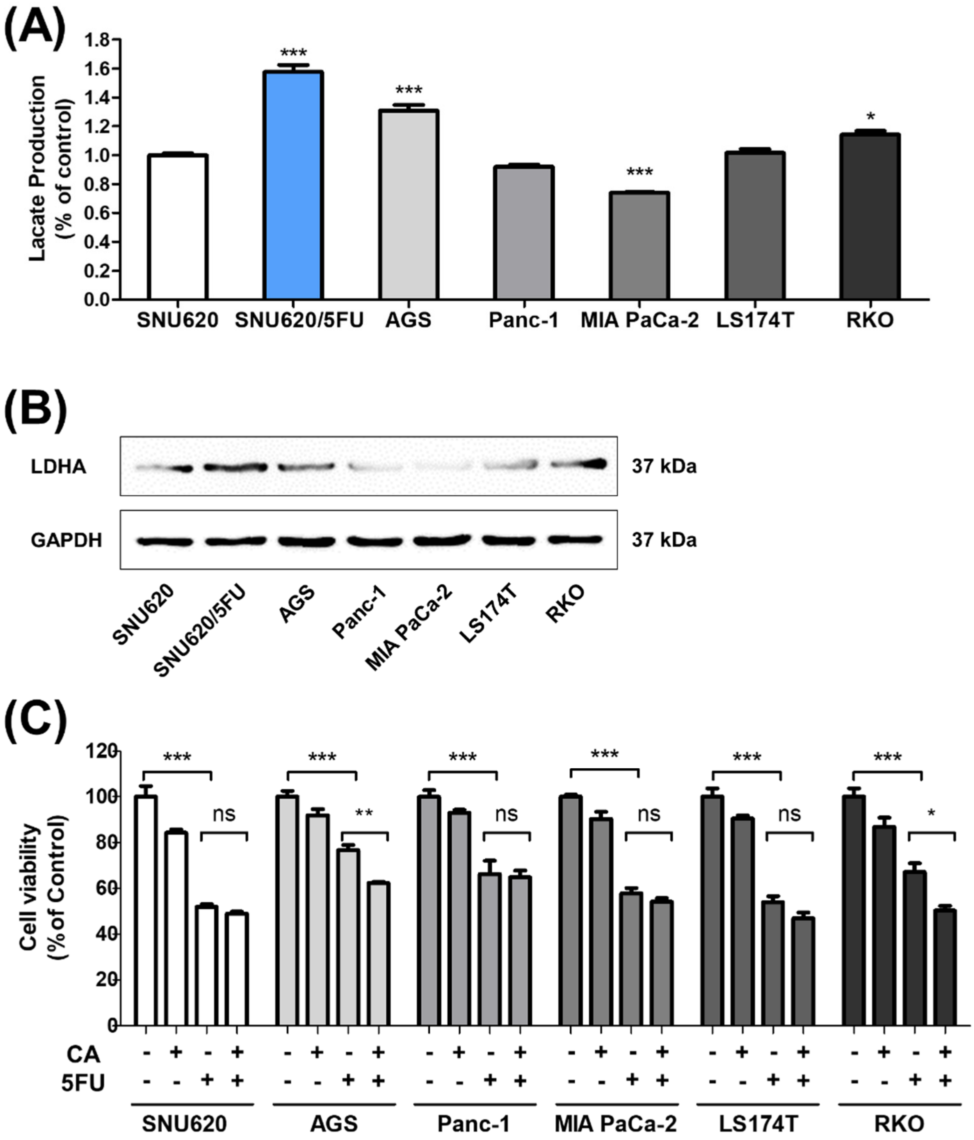

Supplementary Figure S10. Measurement of glycolytic phenotypes and cell viability in various cancer cells. **(A, B)** Lactate production and LDHA expression were measured in SNU620, SNU620/5FU, AGS, Panc-1, MIA PaCa-2, LS174T, and RKO cells using commercial lactate fluorometric assay kit and western blot assay. **(C)** Cell viabilities were evaluated in SNU620, AGS, Panc-1, MIA PaCa-2, LS174T, and RKO cells following catechin (10 μM) and 5FU (10 μM) treatments. After 48 h, cell viability was analyzed via MTT assay. The results are shown as mean ± SEM. * $p < 0.05$, ** $p < 0.01$, and *** $p < 0.001$ compared to the control.

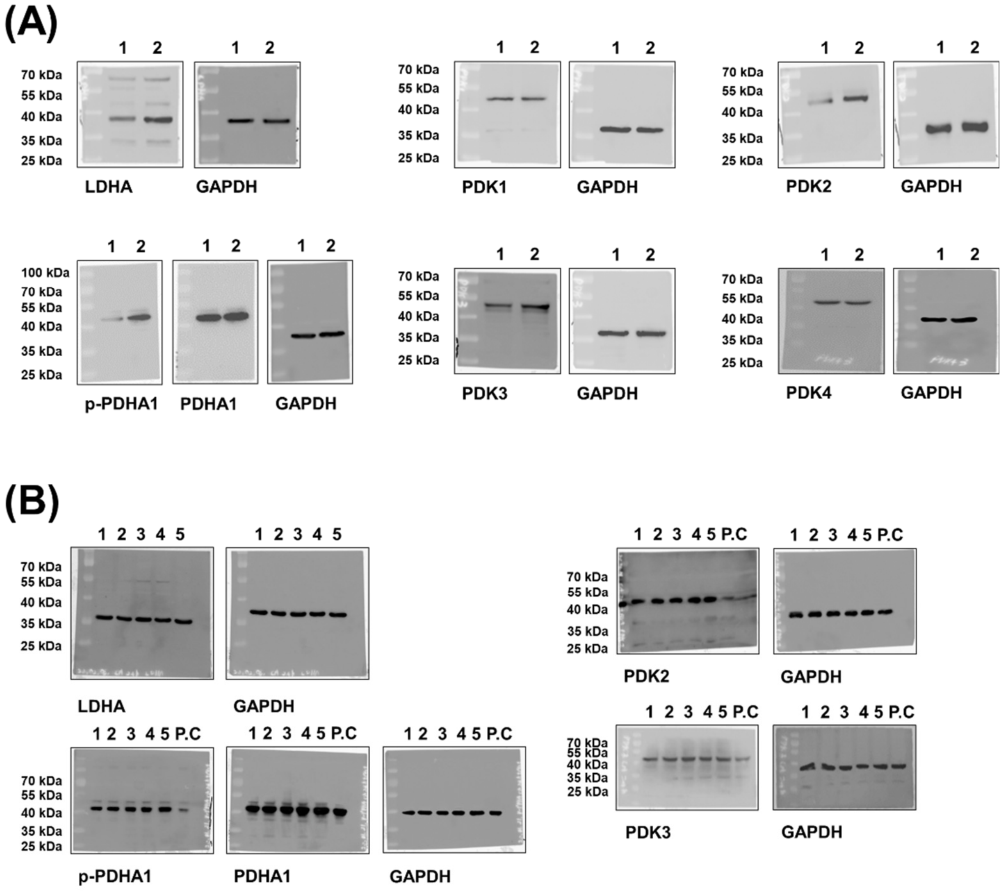
(A)
1 2
70 kDa
55 kDa
40 kDa
35 kDa
25 kDa
LDHA
GAPDH
PDK1
PDK2
100 kDa
p-PDHA1
PDHA1
PDK3
PDK4
(B)
1 2 3 4 5
LDHA
GAPDH
1 2 3 4 5 P.C
PDK2
p-PDHA1
PDHA1
PDK3

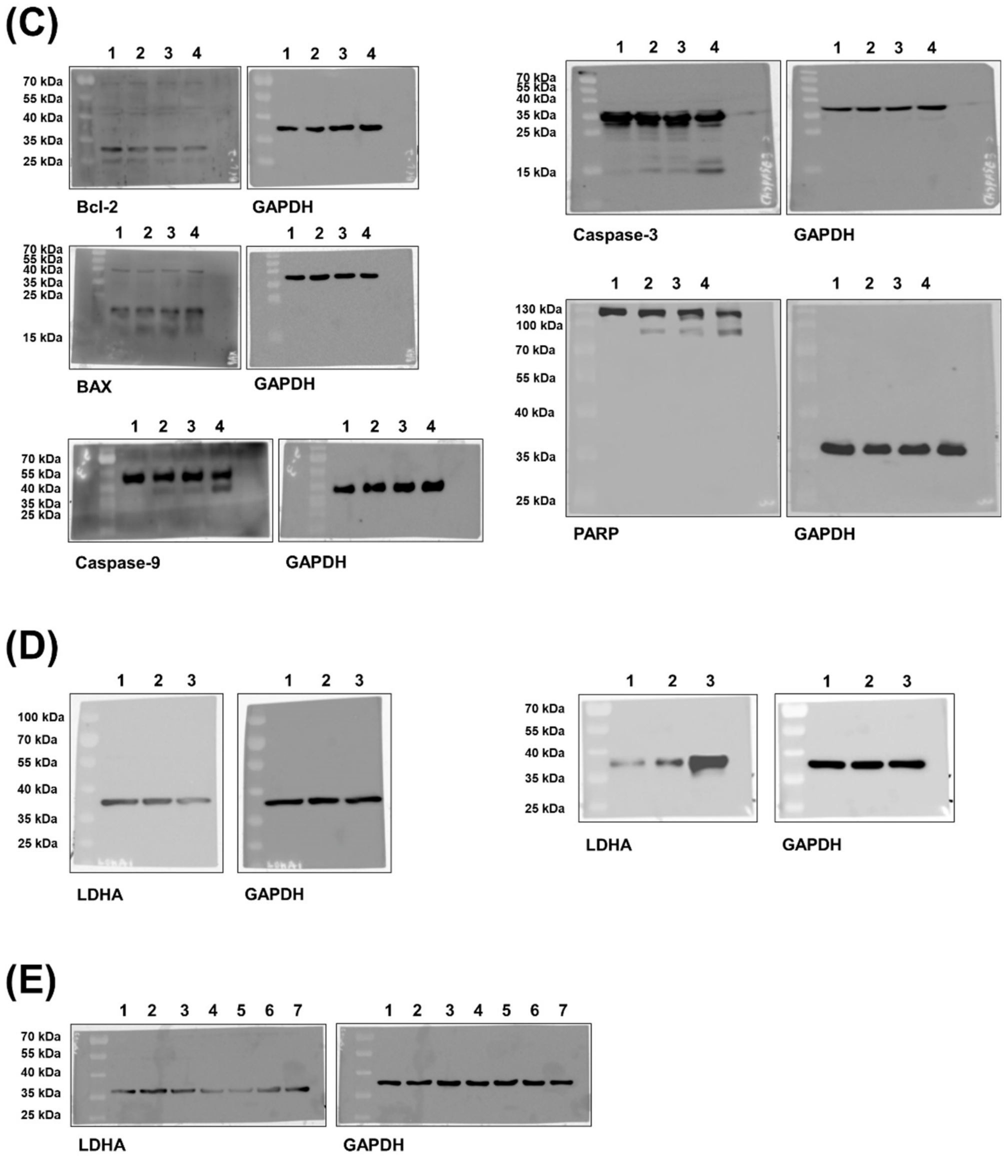

Supplementary Figure S11. The raw data of Western blot assay. The figures display all blots used in this study
